# New frontiers in pharmacological treatment of attention-deficit hyperactivity disorder

**DOI:** 10.1007/s00210-025-04328-z

**Published:** 2025-06-06

**Authors:** Ashraf A. Noah, Heba Essam Sedky

**Affiliations:** 1 Alexandria Clinical Research Administration, Directorate of Health Affairs, Egyptian Ministry of Health and Population, Alexandria, Egypt; 2 Department of Pharmacology and Experimental Therapeutics, Medical Research Institute, Alexandria University, Alexandria, Egypt

**Keywords:** ADHD treatment, Stimulant medications, Non-stimulant medications, Neurodevelopmental disorder, Innovative therapies

## Abstract

Attention deficit hyperactivity disorder (ADHD) is a neurodevelopmental disorder characterized by inattention, hyperactivity, and impulsivity. This manuscript reviews the current understanding of ADHD and evaluates treatment approaches, including emerging technologies, to improve outcomes and reduce societal impact. A comprehensive review of the literature was conducted, focusing on stimulant-based therapies, non-stimulant alternatives, and novel therapeutic devices. Clinical efficacy, safety profiles, and advancements in technology-based interventions were analyzed. Stimulant medications, such as methylphenidate and amphetamines, remain the mainstay of ADHD treatment but pose risks of abuse and dependence. Non-stimulant options, including atomoxetine and extended-release α-2 agonists like guanfacine and clonidine, provide alternatives for patients who cannot tolerate stimulants. Emerging therapeutic devices, including the Monarch eTNS System and EndeavorRx, offer innovative drug-free treatment options. The prognosis for ADHD is variable, with some individuals experiencing symptom resolution while others continue to face lifelong challenges. A multidisciplinary approach that integrates pharmacotherapy, behavioral therapy, and novel technology-based interventions holds promise for improving patient outcomes and addressing the societal impact of ADHD.

## Introduction

Attention-deficit/hyperactivity disorder (ADHD) is classified as a neurodevelopmental disorder, characterized by pronounced and developmentally inappropriate levels of inattention and hyperactivity/impulsivity, persisting for over 6 months and manifesting across multiple settings, such as home and school (Faraone and Radonjić 2023).

ADHD is one of the most common psychiatric disorders with a global prevalence range between 5.9 and 12.4% (Al-Wardat et al. 2024). The overall prevalence of ADHD in children and adolescents is 7.6%, with different rates for subtypes: 33.2% for the attention subtype, 30.3% for the hyperactive-impulsive subtype, and 31.4% for the combined subtype (Salari et al. 2023). In the Arab world, a systematic review estimated the prevalence of ADHD among Saudis to be 12.4% (Aljadani et al. 2023). Moreover, previous studies estimated the prevalence of ADHD in Arab Gulf countries, Arab countries, and Africa to range between 5.9 and 10.5% (Almojarthe 2023; Ayano et al. 2020).

Early-onset ADHD, typically diagnosed before age 12, begins in early childhood with symptoms often emerging between ages 3 and 6 and is characterized by impairing symptoms across the core domains of inattention, hyperactivity, and impulsivity, including disorganization, difficulty completing tasks, forgetfulness, and frequent loss of personal items (Singh et al. 2015). To meet diagnostic criteria, symptoms must last at least 6 months, occur in more than one setting, and substantially impair daily functioning (Feldman & Reiff 2014). The impact of ADHD is far-reaching, with potential adverse effects on education, employment, social interactions, and overall quality of life (Agarwal et al. 2012). Individuals with ADHD may face increased risk of engaging in dangerous behaviors, experiencing job loss, and struggling with academic achievement (French et al. 2023).

The underlying pathology of ADHD involves dysregulation of executive functions, largely associated with frontal lobe activity (Petrovic and Castellanos 2016). As a result, those with ADHD often struggle with attention, decision-making, and emotional regulation (Humphreys et al. 2016). These difficulties can lead to frustration, impulsivity, and challenges in social interactions, sometimes leading to a stigmatizing “troublemaker” label (Harborne 2001).

Despite the challenges, there are evidence-based treatments available, including pharmacotherapy and behavioral interventions (Caye et al. 2019). Early diagnosis and comprehensive treatment strategies are essential in managing symptoms and mitigating long-term consequences, thereby improving outcomes for individuals with ADHD (Katzman et al. 2017).

## ADHD pathophysiology

**Fig. 1:**
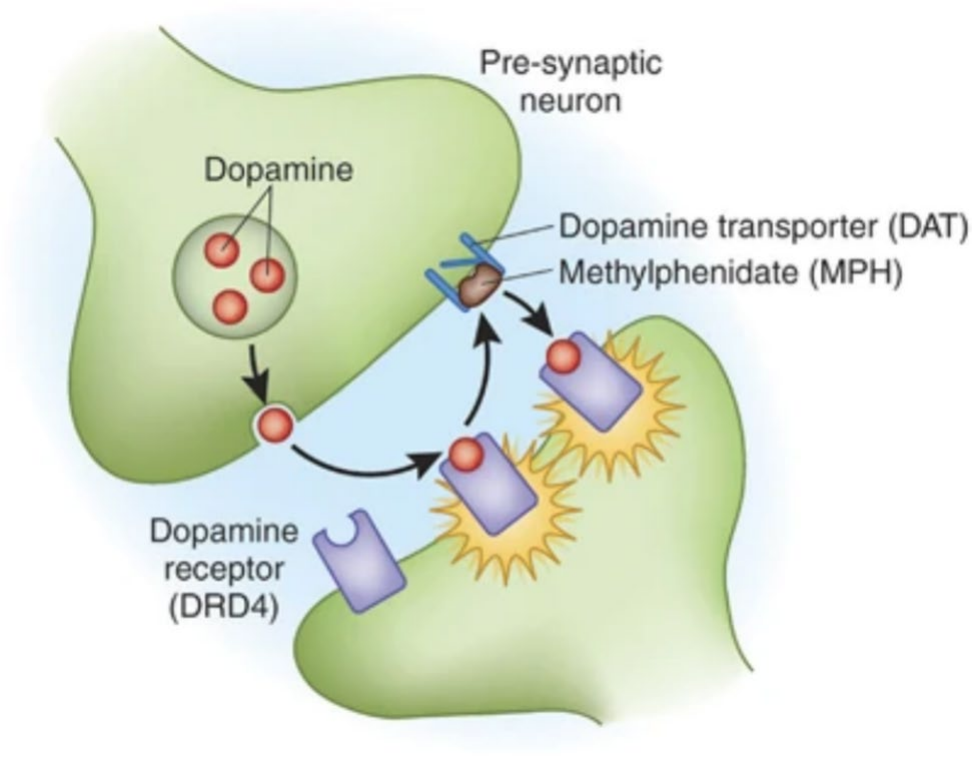
Schematic representation of DA regulation. Adapted from Mehta, T.R. et al. (Bush 2010)

ADHD has been linked to a complex interplay of genetic, neurological, and environmental factors, yet the precise pathophysiology of the disorder remains incompletely understood (Cortese 2012). One of the most consistent findings is the dysregulation of neurotransmitters, specifically dopamine (DA) and norepinephrine (NE), which are crucial for maintaining attentional control and impulse regulation (Mehta et al. 2019) (Fig. 1).

Dopaminergic neurons release DA into the synapse, where it communicates with post-synaptic neurons via specific receptors, like DRD4. Following this signaling, DA is reabsorbed into the presynaptic neuron through the dopamine transporter (DAT). Stimulants such as methylphenidate can inhibit DAT, leading to increased levels of DA in the extracellular space to correct the imbalance of DA encountered in ADHD.

These neurotransmitters exert their effects through G-protein coupled receptors (GPCRs), and their activity is modulated by various transporters (Sahin et al. 2018). Both GPCRs and neurotransmitter transporters represent key targets for pharmacological intervention in ADHD (Huang et al. 2019). Alterations in their function can contribute to the characteristic symptoms of the disorder (Cortese 2012).

Structural and functional abnormalities have also been identified in specific brain regions, including the prefrontal cortex (PFC), caudate nucleus, basal ganglia, anterior cingulate cortex, and cerebellum (Qiu et al. 2011). Functional MRI studies reveal that these brain structures show atypical development and altered activation patterns in individuals with ADHD (Albajara Sáenz et al. 2019). Given that these regions are involved in critical cognitive processes—such as attention, executive function, learning, inhibitory control, emotional regulation, and behavioral responses—their disruption is closely associated with ADHD symptoms like distractibility, impulsivity, forgetfulness, poor planning, and hyperactivity (Depue et al. 2010).

## Diagnosis

ADHD is a condition that is diagnosed through clinical assessment, without the aid of specific laboratory or imaging tests (Brunkhorst-Kanaan et al. 2020). Neuropsychological assessments, while valuable for some purposes, are generally not sensitive enough to diagnose ADHD accurately (Pettersson et al. 2018). Therefore, the diagnosis relies heavily on the patient’s history (Hechtman 2000).

To evaluate a patient for ADHD, clinicians typically use various rating scales and gather information from multiple sources, such as parents and teachers (Dobrean et al. 2021). It is crucial for clinicians to also consider the presence of other disorders that could account for the symptoms, ensuring that the ADHD diagnosis is not made when the symptoms are due to another condition, such as a psychotic or manic episode (Primich & Iennaco 2012).

## ADHD pharmacological treatment

### Stimulants

In the USA, the FDA has approved two classes of stimulant-based therapies for treating ADHD across all age groups: amphetamines and methylphenidate (Weyandt et al. 2014). These stimulants work by interacting with and inhibiting the DAT-1 and the NE transporter (NET), which leads to reduced reuptake of DA and NE, resulting in increased levels of these neurotransmitters in the synapse (Quintero et al. 2022).

Amphetamine has a unique mechanism among stimulants, as it can enter the presynaptic terminal through DAT-1 and NET, allowing it to release stored neurotransmitters (Robertson et al. 2009).

Additionally, both amphetamines and methylphenidate inhibit monoamine oxidase, the enzyme responsible for metabolizing catecholamines, with amphetamine exhibiting greater potency in this regard (Feinberg 2004).

Overall, the effects of both types of stimulants lead to an increase in synaptic levels of DA and NE (Arnsten 2006). The slight differences in their mechanisms of action help explain why some patients who do not respond to one stimulant may have a more favorable response to the other (Pliszka 2005).

#### Methylphenidate

Methylphenidate (MPH), due to its two chiral centers, can exist as two racemic pairs, each containing two enantiomers: the d,l-erythro pair and the d,l-threo pair (Smith 2021). Although both enantiomers of the threo pair exhibit stimulant properties, the l-enantiomer is generally considered inconsequential because it undergoes significant first-pass metabolism, limiting its bioavailability (Jaeschke et al. 2021).

The therapeutic effects of MPH are closely linked to its impact on DA levels in the brain, notably in regions like the ventral striatum, PFC, and temporal cortex (Engert & Pruessner 2008). MPH administration has been shown to normalize activity in frontocingulate networks and striatal areas, while also enhancing frontoparietal connectivity, which is essential for working memory (Rubia et al. 2011).

The long-term effects of psychostimulants like MPH are complex, with research on chronic MPH use yielding conflicting outcomes—some studies indicate increased sensitivity to the drug’s locomotor effects, while others report tolerance or no significant changes, possibly due to variations in dosage, administration methods, age, or treatment history; however, recent studies with ADHD animal models suggest that chronic MPH use may induce brain plasticity, impacting genes and proteins linked to neuroplasticity and catecholamine function, underscoring the need for further research to understand the broader implications for adult ADHD (Contreras et al. 2022; Simchon Tenenbaum et al. 2015).

Furthermore, other neurobiological changes associated with MPH administration include improved error detection linked to activation in the dorsal anterior cingulate cortex and the inferior parietal lobe, as well as optimized reaction speed correlated with increased activity in the pre-motor cortex (Moeller et al. 2014). Beyond these specific changes, MPH has been shown to increase activity in several other brain regions, including the caudate nucleus, cerebellum, midbrain, substantia nigra, and thalamus (Mueller et al. 2014). A study also reported enhanced functional connectivity in the anterior cingulate, ventrolateral PFC, and precuneus, which has implications for working memory performance (Sala-Llonch et al. 2012).

Despite some evidence suggesting that amphetamines may have slightly better efficacy, MPH is often preferred due to its slower rate of uptake and clearance, which reduces the risk of dependency and potential harm (Childress et al. 2019). Additionally, MPH tends to increase DA levels in the brain primarily when there is active DA release, such as during cognitively demanding tasks, thus reducing the potential for abuse (Clemow 2017).

Another advantage of MPH is its metabolism through hepatic and peripheral esterases, which makes it less prone to interactions with other drugs that are metabolized by the cytochrome P450 (CYP) enzyme system (Williard et al. 2007). This characteristic further reduces the risk of drug interactions and adds to the overall safety profile of MPH in comparison to amphetamines (Williard et al. 2007).

#### Amphetamine salts

Amphetamines, consisting of d- and l-optical isomers, are metabolized by various cytochrome P450 (CYP) enzymes, including CYP1 A2, CYP2 C9, CYP2D6, and CYP3 A4 leading to a high potential for drug interactions (Sharma and Couture 2014). The CYP2D6 enzyme also has genetic polymorphisms, resulting in “slow” and “fast” metabolizers among individuals (Kane 2021). As a result, certain populations, such as Latinos, African Americans, and approximately 7% of Caucasians, may exhibit elevated amphetamine concentrations due to slower metabolism (Dopheide and Pliszka 2009). A cautious and gradual titration of the amphetamine dose, like the recommended approach for methylphenidate, can help minimize the risk of adverse effects and toxicity in slow metabolizers (Duong et al. 2012).

Amphetamines have an inherent risk for abuse, dependence, and neurotoxicity if not used according to prescription guidelines (Sanchez-Ramos 2015). To mitigate these risks, the prodrug lisdexamfetamine has been developed with a slower rate of release and absorption, which can reduce the potential for euphoria, thereby lowering the risk of misuse (Ermer et al. 2016). This pharmacokinetic property makes lisdexamfetamine a potentially safer option among amphetamines for ADHD treatment (Madaan et al. 2013).

### Nonstimulants

While stimulant medications are generally the first choice for treating ADHD, they may not be appropriate for nearly 30% of patients (Arnsten 2006). This might be due to lack of response or partial response, intolerance to side effects (such as insomnia), the presence of medical comorbidities like psychiatric, cardiovascular, or tic disorders, or concerns from families about the use of controlled substances (Dittmann et al. 2018). In these cases, nonstimulant medications offer an alternative, either as a replacement for stimulants or as adjunctive therapy to manage ADHD (Silver 1999). The FDA has approved three nonstimulant medications for treating ADHD: atomoxetine (Strattera), and the extended-release α−2 agonist clonidine (Kapvay) and guanfacine (Intuniv) (Childress and Berry 2012).

Nonstimulants are generally believed to have a less potent effect compared to stimulants, but direct, well-controlled comparisons between these drug classes are lacking (Upadhyaya et al. 2013). The perception of greater efficacy for stimulants, particularly methylphenidate, over nonstimulants like atomoxetine, is largely derived from meta-analyses and clinical experience rather than head-to-head clinical trials (Elliott et al. 2020). As such, while stimulants remain the preferred option for many patients, nonstimulants provide an important treatment alternative for those who cannot use or do not respond well to stimulant therapy (Schulz et al. 2012).

#### Atomoxetine (strattera)

Atomoxetine acts by increasing the availability of NE and DA in the PFC, primarily through the selective inhibition of their presynaptic transporters (Bymaster et al. 2002). It also serves as an NMDA receptor antagonist, influencing glutamatergic transmission (Ludolph et al. 2010). In contrast to stimulant medications, atomoxetine has limited effects on the striatum and carries a lower risk of abuse, partly because its full therapeutic effect may take 4–6 weeks to develop (Fu et al. 2022).

Neurobiological changes associated with atomoxetine use include increased regional cerebral blood flow in the cerebellar cortex and decreased blood flow in the midbrain, substantia nigra, and thalamus (Cortese 2012). Enhanced inhibitory control observed after atomoxetine treatment has been linked to increased activation in the right inferior frontal gyrus (Chamberlain et al. 2009).

Both amphetamine and atomoxetine are metabolized by the CYP2D6 enzyme, so it is advised to gradually titrate atomoxetine dosage to reduce the risk of toxicity, particularly in patients who are known to be CYP2D6 slow metabolizers (Dean 2020).

While atomoxetine, like stimulants, is contraindicated in patients with clinically significant arrhythmias, the presence of stable congenital or structural cardiac anomalies with preserved cardiac function does not constitute an absolute contraindication, provided that individualized risk–benefit assessment and appropriate cardiovascular monitoring are undertaken (Topriceanu et al. 2022).

#### α−2 Agonists

Immediate-release α−2 agonists, such as guanfacine and clonidine, were originally FDA-approved for treating hypertension but have been used off-label for managing ADHD (Connor et al. 2014). Recently, extended-release forms of these drugs have gained FDA approval for treating ADHD in children and adolescents (Bello 2015). Although their efficacy may not be as high as stimulant medications, these α−2 agonists could be as effective as behavioral therapy, suggesting that behavioral interventions should be considered before using these agents (Harstad et al. 2021).

Guanfacine works by inhibiting cyclic AMP, which leads to the closing of hyperpolarization-activated cyclic nucleotide-gated (HCN) channels, thereby enhancing functional connectivity in the PFC (Huss et al. 2016). Blocking or reducing HCN1 channels in the PFC is thought to improve working memory. Guanfacine has been shown to increase activation in the dorsolateral PFC, and because glutamatergic synapses in this region contain inhibitory α2 A-adrenoceptors, it is speculated that both guanfacine and clonidine could effectively treat ADHD by reducing presynaptic glutamate release in the PFC (Arnsten 2011).

Guanfacine and clonidine target both presynaptic and postsynaptic α2 receptors on neuronal cells, but guanfacine’s greater selectivity for postsynaptic α2 A receptors—believed to be crucial for alleviating ADHD symptoms—along with its longer half-life, suggests it might be more effective and associated with reduced sedation and dizziness compared to clonidine (Arnsten 2010).

#### Bupropion

Bupropion, an FDA-approved antidepressant and smoking cessation medication, is used off-label to treat ADHD based on evidence from various studies, and while it shares a similar mechanism with stimulants and atomoxetine by inhibiting the reuptake of DA and NE, it carries a lower risk of abuse and dependence (Reimherr et al. 2005).

Bupropion generally has a quicker onset of action than atomoxetine or α−2 agonists, often showing therapeutic benefits within two weeks and demonstrating comparable efficacy in treating ADHD; however, it carries a heightened risk of seizures and should be avoided in patients with a history of seizures or eating disorders, with side effects usually being mild and manageable through divided dosing (Heal et al. 2009).

#### Tricyclic antidepressants

Tricyclic antidepressants (TCAs) were once commonly used off-label for ADHD treatment in monotherapy, favored for their long duration of action, absence of abuse potential, and ability to address co-occurring depression and tics, but they have since declined in popularity due to their significant cardiovascular, neurological, and anticholinergic side effects, interactions with other medications, and generally lower efficacy compared to stimulants (Otasowie et al. 2014).

TCAs are typically used for ADHD only when patients do not respond to stimulants or other alternatives, cannot tolerate those medications, or have specific comorbidities like tics or depression that could worsen with stimulant use, with imipramine, desipramine, and nortriptyline being the most common TCAs for ADHD—though desipramine is least preferred due to instances of sudden cardiac death, requiring dose adjustments for hepatic impairment and an electrocardiogram to ensure cardiac safety before and after any dosage increase (Park et al. 2014).

TCAs should be gradually titrated to enhance tolerability and minimize toxicity risks—particularly in slow metabolizers due to their CYP2D6 metabolism—and must be tapered off slowly when discontinuing to avoid withdrawal symptoms, emphasizing the need for careful risk assessment and thorough monitoring despite their utility as an alternative for specific cases (Burke and Preskorn 2004).

### Novel therapeutic modalities approved by the FDA

#### Novel drugs

New medications for ADHD have been developed in recent years, offering a diverse range of treatment options that go beyond traditional stimulants (Drechsler et al. 2020). These novel therapies offer unique benefits like non-stimulant mechanisms of action, innovative release systems, and transdermal delivery, representing significant progress in ADHD treatment by expanding the range of effective options for children and adults (Nazarova et al. 2022). In the table below is a summary of key ADHD medications, detailing their applications, approval status, and distinctive features (Table 1).

**Table 1:** Novel ADHD medications

Medication name	Type	Approval date	Description
Viloxazine (Qelbree®)	Non-stimulant ADHD medication	2021 for children over six and 2022 for adults	Qelbree is a non-stimulant ADHD medication approved for pediatric use that uniquely modulates both NE and serotonin (5HT) signaling, representing the first agent with this dual mechanism of action, distinct from earlier non-stimulants such as atomoxetine and α2-adrenergic agonists (Price & Price 2023)
Serdexmethylphenidate and Dexmethylphenidate (Azstarys®)	Central nervous system stimulant	2021 for patients aged six and older	Azstarys, a stimulant for ADHD in children over six years old taken once daily, contains serdexmethylphenidate, a prodrug of d-methylphenidate; it is classified as a Schedule II controlled substance (Kollins et al. 2021)
Methylphenidate HCl (Jornay PM®)	Stimulant ADHD medication	2018 for patients aged six and older	Jornay PM, a delayed-release/extended-release methylphenidate formulation administered in the evening, features a delayed onset of 8–10 h followed by a prolonged release lasting 10–12 h, delivering a gradual therapeutic effect that begins the next morning and continues throughout the day to manage ADHD symptoms and improve early morning functioning (Romba & Singh 2025)
Dextroamphetamine (Xelstrym®)	Stimulant ADHD medication	March 2022 for children ages 6–17 and adults 18 and older	Xelstrym, the first FDA-approved transdermal amphetamine patch for ADHD, provides a once-daily treatment with consistent medication release, serving as an alternative to oral ADHD medications and potentially reducing the stigma of taking pills in public (Larkin 2022)
Vortioxetine (Trintellix®)	Antidepressant	September 30, 2013, for major depressive disorder (off label for ADHD)	Vortioxetine is a 5HT modulator and stimulator. It works by inhibiting the reuptake of 5HT and modulating 5HT receptor activity, which helps to increase 5HT levels in the brain and improve mood (Sowa-Kućma et al. 2017)
Droxidopa (Northera®)	Prodrug of NE	February 18, 2014	Droxidopa is converted to NE, a neurotransmitter that helps to increase blood pressure by inducing vasoconstriction and increasing heart rate. It primarily treats neurogenic orthostatic hypotension (NOH) but can also be used off-label for ADHD symptoms (Palma & Kaufmann 2023)

### ADHD devices

Recently, two devices received FDA approval for treating ADHD: the Monarch eTNS System and EndeavorRx (Nazarova et al. 2022). These innovative approaches, which offer non-pharmacological alternatives for managing ADHD symptoms, are listed in the table below (Table 2) (Camp et al. 2021).

**Table 2:** ADHD FDA-approved devices

Device name	Type	Approval date	Description
Monarch eTNS System	Non-invasive electronic device	2019	The Monarch eTNS System, the first FDA-approved device-based treatment for ADHD, uses low-level electrical stimulation on the trigeminal nerve to potentially boost activity in brain regions tied to executive functions, like the anterior cingulate and frontal gyri, though the exact mechanism is unclear; it's approved as a monotherapy for children aged 7–12 with caregiver supervision (Rubia et al. 2024)
EndeavorRx	Game-based digital therapeutic	June 2020	EndeavorRx is the first FDA-approved game-based digital therapeutic for ADHD, targeting children aged 8–12 with interactive graphics and rewards to stimulate brain regions tied to attention, inhibitory control, and working memory, with studies showing it can result in measurable cognitive gains (Oh et al. 2024)

*eTNS* external Trigeminal Nerve Stimulation

These devices indicate a promising trend towards drug-free alternatives in ADHD treatment (Nazarova et al. 2022). They offer unique methods of addressing ADHD symptoms, potentially reducing the need for traditional pharmacological interventions (Teruel et al. 2017). Their development underscores a growing interest in exploring new avenues for ADHD management through technology and innovative therapeutic approaches (Tiitto et al. 2017).

#### New medications under clinical trials

Research into new agents to treat ADHD or repurpose existing drugs is a worldwide endeavor aimed at finding more effective treatments with fewer side effects (Mechler et al. 2022). In Table 3, we highlight some of the leading current efforts to develop new medications for ADHD treatment, as documented in the United States National Institutes of Health’s clinical trials registry over the past 3 years, available at www.clinicaltrials.gov.

**Table 3:** New ADHD medications under development

Interventions	Drug type	Phase	Sponsor	NCT number
NRCT-101-SR	The agent acts on glutamatergic synapses to increase synaptic density, function, and plasticity in the prefrontal cortex and hippocampus	2/3	Neurocentria, Inc	NCT05683249
Solriamfetol (75–150 mg)	DA and NE modulator	2/3	Massachusetts General Hospital	NCT04839562
Centanafadine hydrochloride	A triple reuptake inhibitor that targets NE, DA, and 5HT transporters	3	Otsuka Pharmaceutical Development & Commercialization, Inc	NCT05257265
OPC-34712	Known as “Brexpiprazole,” it is a partial agonist for 5-HT1 A and D2 receptors, and an antagonist of 5-HT2 A receptors	2	Otsuka Pharmaceutical Development & Commercialization, Inc	NCT01074294
Carbidopa	Carbidopa is a medication commonly used in combination with levodopa to prevent the conversion of levodopa to DA outside the brain	2	Chelsea Therapeutics	NCT00983814
SPN-812	Extended release Viloxazine primarily acts as a NE reuptake inhibitor	3	Supernus Pharmaceuticals, Inc	NCT02736656
SPD465	It is an extended-release formulation of mixed amphetamine salts	2	Takeda (Shire)	NCT00928148
SPD489	Also known as Lisdexamfetamine, it is a prodrug that is converted to dextroamphetamine in the bloodstream after oral administration	4	Takeda (Shire)	NCT00877487
CTx-1301	It is a controlled-release formulation of dexmethylphenidate that inhibits NE and DA reuptake	3	Cingulate Therapeutics	NCT05631626
ABT-089	An agent that belongs to a class of drugs known as α4β2 nicotinic acetylcholine receptor agonists	2	AbbVie (prior sponsor, Abbott)	NCT00528697
MM-120	It is a selective 5HT and NE reuptake inhibitor	2	Mind Medicine, Inc	NCT05200936
PDC-1421	It is a NE plasma membrane transport protein inhibitor	2	BioLite, Inc	NCT02699086

*NCT* National Clinical Trial number

## Prognosis

The prognosis for ADHD varies with the age of symptom onset, with symptoms frequently persisting into adolescence and affecting social and academic aspects of life; around 40% of teenagers with ADHD continue to have symptoms, and 25% might develop comorbid antisocial disorders (Caye et al. 2016).

Over time, ADHD symptoms tend to decrease by about 50% as individuals transition into adulthood, with half of patients effectively “growing out” of ADHD—especially with treatment—while another 25% no longer require ongoing therapy, possibly due to stimulants fostering frontal lobe development and adults opting for careers with lower attention demands, allowing many adults with ADHD to achieve their educational and professional goals (Treuer et al. 2017).

Treatment of ADHD has been linked to improved symptoms of oppositional defiant disorder and conduct disorder, along with a lower risk of substance use, while untreated ADHD can lead to long-term problems such as persistent dysfunction, an elevated risk of car accidents, and greater substance use, underscoring the importance of effective treatment to mitigate these risks and enhance quality of life for those with ADHD (Shaw et al. 2012).

## Future drug targets for ADHD

Recent ADHD research has delved into neurobiology, including neuropeptides, which are core signaling molecules in the brain and nervous system (Branca and Bortolato 2024). Additionally, there is growing interest in the neuroendocrine system, particularly the role of neurosteroids such as dehydroepiandrosterone (DHEA) and its sulfated form, DHEA-S. Evidence suggests that lower blood levels of DHEA and DHEA-S correlate with more severe hyperactivity-impulsivity symptoms in children with ADHD (Şahin et al. 2022). Moreover, methylphenidate elevates allopregnanolone in inattentive ADHD patients without depression but diminishes/reverses in hyperactive-impulsive subtypes or those with depression, suggesting neurosteroid levels may guide personalized ADHD treatment (Molina-Carballo et al. 2014).

Beyond the traditional dopaminergic and noradrenergic systems, researchers are exploring other pharmacological targets, like the cannabinoid system (Dawson and Persad 2021). A trial with low-dose cannabinoids for ADHD core symptoms reported moderate-to-large effects, suggesting that cannabinoid-based treatments could be a promising area for future drug development (Cooper et al. 2017).

Despite these advancements, pharmacological treatment alone may not be enough to manage ADHD symptoms and achieve optimal functional outcomes (Bolea-Alamañac et al. 2014). About 30% of patients with ADHD do not respond sufficiently to stimulants or experience adverse effects, raising concerns about tolerance, while issues surrounding stimulant misuse and diversion persist, underscoring the need for more comprehensive treatment approaches and continued research into safer and more effective therapies (Childress and Sallee 2014).

## Conclusion

ADHD remains a complex and multifaceted condition that affects a significant portion of the population across age groups, with implications for both individual well-being and broader societal outcomes. As we have discussed, ADHD is characterized by neurodevelopmental disruptions leading to severe inattention, hyperactivity, and impulsivity that manifest in multiple areas of life. This disorder requires a comprehensive approach to diagnosis and treatment, reflecting its varied presentations and significant impact on day-to-day functioning.

The understanding of ADHD’s pathophysiology has evolved, suggesting a mix of genetic, neurological, and environmental influences. Neuroimaging studies have revealed alterations in brain structures and neurotransmitter dysregulation, particularly in DA and NE pathways. However, despite these insights, the precise mechanisms underlying ADHD remain elusive, emphasizing the need for continued research to better understand and address the condition.

Treatment options for ADHD are extensive, with stimulant-based therapies being the most common first-line agents. Methylphenidate and amphetamines have proven effective in managing ADHD symptoms, yet they carry risks of abuse and dependence, necessitating cautious use and monitoring. Non-stimulant medications, such as atomoxetine, and extended-release α−2 agonists like guanfacine and clonidine, offer alternative treatment pathways for those who cannot tolerate stimulants or require adjunctive therapy. Novel therapeutic devices like the Monarch eTNS System and EndeavorRx represent innovative, drug-free options, highlighting a shift toward technology-based interventions in ADHD treatment.

Emerging therapeutic targets, including neurosteroids and cannabinoids, indicate a growing exploration of alternative pathways for ADHD treatment. While these new approaches hold promise, they require further investigation to determine their safety, efficacy, and long-term impacts. Additionally, the potential disease-modifying effects of existing treatments, such as atomoxetine, suggest that pharmacotherapy might lead to lasting changes in brain function, providing new avenues for improving outcomes in ADHD.

The future of ADHD treatment lies in a multidisciplinary approach that integrates pharmacotherapy, behavioral interventions, and innovative technologies. Collaboration among clinicians, researchers, patients, and families is crucial to develop more effective, individualized, and sustainable treatment strategies. With continued research and innovation, we can aspire to improve the quality of life for individuals with ADHD and reduce the broader societal impact of this challenging disorder.

## Data Availability

All source data for this work (or generated in this study) are available upon reasonable request.

## Abbreviations

*ADHD*: Attention-deficit/hyperactivity disorder
*DA*: Norepinephrine
*DRD4*: Dopamine receptor D4
*DAT*: Dopamine transporter
*GPCRs*: G-protein coupled receptors
*PFC*: Prefrontal cortex
*MPH*: Methylphenidate
*TCAs*: Tricyclic antidepressants
*NMDA*: N-Methyl-D-Aspartate
*5HT*: 5-hydroxytryptamine (serotonin)
*DHEA*: Dehydroepiandrosterone
*DHEA-S*: Dehydroepiandrosterone sulfate
